# Demographics and Academic Productivity of Cardiothoracic Imaging Fellowship Program Directors in the United States: A Cross-Sectional Review

**DOI:** 10.7759/cureus.26855

**Published:** 2022-07-14

**Authors:** Zachary D Zippi, Benjamin I Schachner, Nathan Vanderveer-Harris, Nicholas Jaeger, Michael Zalkind, Justin Stowell, Patricia J Mergo

**Affiliations:** 1 Office of Medical Education, Florida International University, Herbert Wertheim College of Medicine, Miami, USA; 2 Office of Medical Education, University of Miami Miller School of Medicine, Miami, USA; 3 Department of Internal Medicine, Naples Community Hospital Healthcare System, Naples, USA; 4 Department of Radiology, Mayo Clinic, Jacksonville, USA

**Keywords:** diagnostic radiology, fellowship director, cardiothoracic imaging, education, leadership

## Abstract

Background

In this study, we aimed to assess current demographics, measures of academic productivity, and other objective leadership characteristics among United States cardiothoracic imaging fellowship directors (FDs).

Methodology

A survey was sent to active members listed in the Society of Thoracic Radiology Cardiothoracic Imaging Fellowship Directory. Demographic, post-graduate training, and scholarly activity data were collected, including, but not limited to, age, sex, residency and fellowship training institutions, time since training completion until FD, length of time as FD, and Hirsch-index (h-index) to measure research activity.

Results

We identified 53 FDs from 50 cardiothoracic imaging fellowship programs. Of these, 31 (58.5%) were male and 22 (41.5%) were female with an average age of 48.5 years (standard deviation (SD) = 8.4, range = 35-67). There was no statistically significant difference between the mean age of male and female FDs (47.5 vs 50.2 years, p = 0.2811). The mean age of appointment to the FD role was 41.8 years. On average, FDs graduated from residency in 2005 and 2007 for fellowships. Most attended allopathic medical schools (52/53, 98.1%). The average Scopus h-index was 15.7 (SD = 17.4). Gender-wise comparison of mean h-indices revealed 16.2 for males and 15 for females, with no statistically significant difference between the two groups (p = 0.81). Ten (18.9%) FDs and 20 (37.7%) FDs were at the same location they completed residency and fellowship training, respectively.

Conclusions

This cross-sectional study shows the present demographics within the cardiothoracic radiology FD position. This field of radiology is observed to have FDs with research productivity that is comparable with other medical specialties. Some radiology residency and fellowship programs were shown to produce more FDs than others; however, we were not able to identify causality. Program directors appear to be selected from a familiar pool of applicants, and ultimately FDs are being replaced by individuals with similar distinctions. Overall, this research into cardiothoracic radiology FDs demographics and research productivity can add to the current body of literature on FDs in various medical specialties. It is important to continue to reflect on medical leadership as the field continues to advance.

## Introduction

Cardiothoracic radiology is a subspecialty of diagnostic radiology. It consists of the interpretation of imaging and interventional procedures of the pleura, mediastinum, lungs, chest wall, heart, pericardium, and thoracic vasculature [1]. The field of cardiothoracic radiology is a rapidly growing field of radiology, with twice as many radiologists completing fellowships in the last five years than in the previous five years [2]. The large expansion of fellowship-trained radiologists is secondary to the development of numerous fellowship training programs.

Academic physicians can have an immense impact on not only the training and career trajectory of the next generation of physicians but also on the practice of medicine itself. Fellowship directors (FDs) hold the potential to shape the future of the fellow by ensuring that their fellowship exposure provides state-of-the-art training in imaging techniques and diagnostic assessment as a foundation for successful careers. They provide trainees with the confidence, mentorship, and knowledge necessary to practice at the highest standards of care. Furthermore, they conduct research in advancing diagnostic and therapeutic knowledge, improving protocols, and our understanding of the practice. These FD roles encompass responsibilities that range from research production, institutional and national academic society engagement, clinical duties, teaching, and innovation.

Only three recent studies have examined the demographic trends among radiology director positions [3-5]. Moreover, other studies examining FDs in different subspecialties such as orthopedic surgery, ophthalmology, and cardiothoracic surgery were published recently [6-10]. In all of these surgical subspecialties, there were a larger number of male directors compared to females. A study of sports medicine orthopedic surgeons observed a disproportionate proportion of male FDs (97.5%) with an average age of 56 years [9]. Ophthalmology showed a similar distribution with the majority being males (72%) with an average age of 50 years [7]. In relation to our focus on radiology, a similar trend was presented by Purushothaman et al., who found a majority of radiology residency program directors to be male (70.6%) with an average age of 47 years [3]. Thus, it appears the disproportionate proportion of male FDs seen in surgical specialties extends into the field of radiology.

Hirsch-index (h-index) is an author-level metric that produces a numerical value to attempt to quantify the scientific output of a researcher [11]. H-index, h, is defined as “the number of papers with citation number ≥h.” Among academic radiologists, the h-index has shown a significant relationship with academic rank (i.e., assistant, associate, professor) [12]. The study above further shows that those who wish to climb the academic ladder must do so by being well versed in research. However, this has not been studied among FDs in cardiothoracic radiology. The examination of research productivity in cardiothoracic radiology is important for informing potential fellowship applicants and the next generation of leaders in the field, as well as assessing the quality of educational leadership in the field.

We aim to investigate the trends in demographics, characteristics, and scholarly output currently in cardiothoracic radiology FD within the United States. This cross-sectional study seeks to define the demographics and academic productivity of FDs in cardiothoracic radiology.

## Materials and methods

Data source and collection

Fellowships for cardiothoracic radiology were identified through the Society of Thoracic Radiology (STR) fellowship directory on March 30, 2021 [13]. The STR website provided hyperlinks directly to each of the respective cardiothoracic fellowships. This generated a total of 57 programs, of which 50 were included in this study, while Canadian programs (n = 7) were excluded. All FDs were identified through their university or hospital fellowship information webpage. For fellowship programs that had co-directors, both were included in the analysis, but assistant directors were excluded. The collection of professional education, residency, residency year, fellowship, and fellowship year was obtained through respective institutional biographies, Doximity (Doximity.com, Doximity Inc., San Francisco, CA), and curriculum vitae (CV). The information was cross-referenced between these two platforms when applicable. The age of each FD was established using Healthgrades (Healthgrades Operating Company Inc, Denver, CO). Emails and questionnaires were sent directly to FDs to acquire their CVs, which were used to assess the year hired by their institution, the year appointed to the FD position, and other demographic information that was not readily available via their respective web pages. CVs were author reviewed for missing information. The demographic information that was of interest included gender, age, past residency training, past fellowship training, year graduated from residency and fellowship, year since graduation to FD appointment, time at institution till FD appointment, and h-index. For any non-responders to surveys, the FD and coordinator of the program were emailed and called twice by the lead author.

Research productivity and impact

The level of scholarly or research productivity and impact was measured by the use of each individual’s h-index. The h-index and publication count were obtained for each FD by searching the full first name and last name within the Scopus database (Elsevier B.V., Waltham, MA, USA).

Institutional loyalty

Institutional loyalty was defined as FDs who trained at either the same residency, fellowship, or both as their current institution where they hold the FD position at. Additionally, the FD’s residency training and fellowship were examined to determine if they trained at the same program for both parts of their training prior to the FD role. This was done to determine the percentage of FDs who were still at or went back to the program they originally trained at.

Statistical analysis

The following data points were gathered for each fellowship leader as set forth by previous leadership papers [6,14,15]: current institution, number of years in the current role, specific role, age, name of residency institution and year of graduation, name of fellowship institution and year of graduation, year hired by current institution, year appointed FD, and Scopus H-index. Mean and standard deviations (SDs) were calculated for all variables stated above. H-index values were grouped by ranges of five with the final group containing all values greater than 30; this was done to display the distribution of indices. Most commonly attended residency programs were obtained and displayed as the top five programs. The top five fellowship programs were displayed, and due to a tie in the number four position, all programs with the value of two were displayed. All of the data were recorded and analyzed using Excel 365 (Microsoft Inc, Redmond, WA, USA). Linear regression was completed using GraphPad QuickCalcs (GraphPad Software Inc, San Diego, CA, USA) and reported as the Pearson coefficient (r) with the respective P-value. Statistical significance was considered at an alpha value of p < 0.05.

## Results

**Figure 1:**
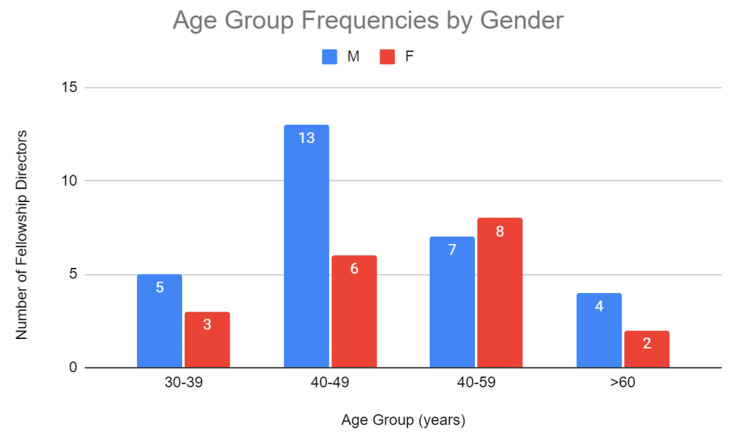
Age group frequencies by gender. A comparison between males and females of the frequency of cardiothoracic radiology fellowship directors distributed by age groups.

A total of 53 FDs were included in the study from 50 fellowship programs, of which 31 (58.5%) were males and 22 (41.5%) were females (Table 1). The response rate via email, questionnaire, or phone call was 27/53 (50.9%). The mean age of FDs was 48.5 years (SD = 8.4, minimum = 35, maximum = 67, n = 48) (Table 1). Mean ages were not statistically significantly different between males and females (47.5 vs 50.2 years, respectively) (p = 0.2811). The mean age at appointment for FDs was 41.8 (SD 7.7, n = 36). The distribution of grouped age frequencies by gender is displayed in Figure 1. The breakdown and distribution of race and ethnicity were not completed as it was not collected on survey responses and the assumption of both would be imprecise.

**Table 1:** Demographics of cardiothoracic radiology fellowship directors. Cardiothoracic radiology fellowship director demographic information including gender distribution, summary age statistics, and average Scopus h-index.

Demographics	Count (%)
Male	31 (58.5%)
Female	22 (41.5%)
	Mean ± standard deviation
Mean age	48.5 ± 8.4 (n = 48)
Mean fellowship director Scopus h-index	15.7 ± 17.4 (n = 53)

Residency and fellowship information was available for all FDs. The only exception was one FD who did not complete a fellowship. The mean duration from residency graduation until appointment to the position of FD was 10.2 years (SD = 7.5, median = 7.5, minimum = 1, maximum = 28, n = 40), while the mean duration from fellowship graduation to the appointment was 8.1 years (SD = 7.4, median = 6, minimum = 0, maximum = 27, n = 39). The average length of duration of employment at the FD’s current institution until the time of this study was 10 years (SD = 8.8, median = 6, minimum = 1, maximum = 39, n = 45), while the average length of time between hire and FD appointment was 4.2 years (SD = 5, minimum = 0, maximum = 24, n = 39). The mean duration of tenure from appointment to the date of this study was 6.6 years (SD = 7.7, median = 3.5, minimum = 0, maximum = 31, n = 40) (Table 2). Institutional loyalty was compared by assessing the FDs’ institutions for residency, fellowship, and FD appointment. FDs with the same residency as their current employment were 10 (18.9%), while 20 (37.7%) FDs maintained loyalty at their same fellowship (Table 3). Only six of 53 (11.32%) FDs were appointed at the same institution where they completed both their residency and fellowship training.

**Table 2:** Education and employment of cardiothoracic radiology fellowship director. A summary statistic table of the career progression of cardiothoracic radiology fellowship directors including residency training, fellowship training, and appointment as fellowship director.

Education and employment progression	Mean score ± standard deviation (n)
Mean calendar year of residency graduation	2005 ± 9.3 (n = 53)
Mean calendar year of fellowship graduation	2007 ± 9.2 (n = 52)
Mean duration from residency graduation to appointment as fellowship director	10.2 ± 7.5 (n = 40)
Mean duration from fellowship graduation to appointment as fellowship director	8.1 ± 7.4 (n = 39)
Mean duration of employment at current institution	10 ± 8.8 (n = 45)
Mean duration of fellowship director tenure	6.6 ± 7.7 (n = 40)
Mean time from hire by the current institution to appointment as fellowship director	4.2 ± 5 (n = 39)
Mean age at appointment as fellowship director	41.8 ± 7.4 (n = 36)

**Table 3:** Fellowship director institutional loyalty. A comparison of institutional loyalty is quantified as a percentage of fellowship directors whose appointment is at the same institution where they completed residency, fellowship, or both. Additionally, shows fellowship directors who did a fellowship at the same program they completed a residency.

Institutional Loyalty	n (%)
Fellowship directors currently working at the same institution as residency training	10 (18.9%)
Fellowship directors currently working at the same institution as fellowship training	20 (37.8%)
Fellowship directors currently working at the same institution as both residency and fellowship training	6 (11.3%)
Fellowship directors who trained at the same institution for residency and fellowship	10 (18.9%)

**Figure 2:**
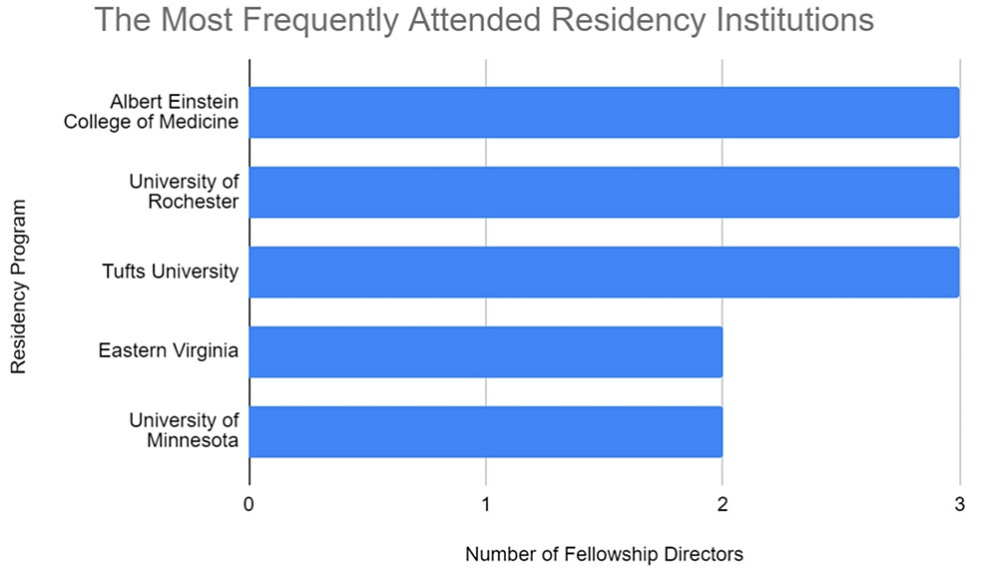
The most frequently attended residency institutions. A summary of the most frequently attended residency institutions of cardiothoracic radiology fellowship directors.

**Figure 3:**
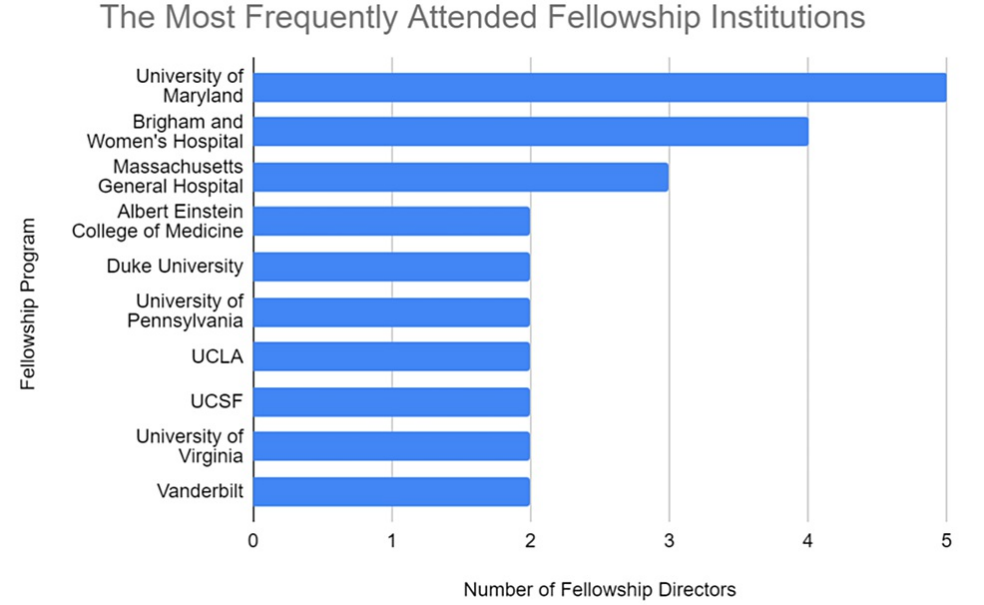
The most frequently attended fellowship institutions. A summary of the most frequently attended fellowship institutions of cardiothoracic radiology fellowship directors. UCLA: University of California, Los Angeles; UCSF: University of California San Francisco

The top five residency programs producing the greatest number of FDs included Montefiore Medical Center (Albert Einstein College of Medicine) (n = 3), University of Rochester Medical Center (n = 3), Lahey Medical Center (Tufts University School of Medicine) (n = 3), Eastern Virginia Medical School (n = 2), and University of Minnesota Medical School (n = 2) (Figure 2). The top three fellowship institutions that produced the most FDs were the University of Maryland Medical Center (n = 5), followed by Harvard-affiliated institutions of Brigham and Women’s Hospital (n = 4) and Massachusetts General Hospital (n = 3). The next seven institutions tied for fourth all produced two FDs and included Montefiore Medical Center (Albert Einstein College of Medicine), Duke University School of Medicine, University of Pennsylvania Medicine, University of California, Los Angeles (UCLA) Health, University of California San Francisco (UCSF) Health, University of Virginia School of Medicine, and Vanderbilt University Medical Center (n = 2) (Figure 3).

**Figure 4:**
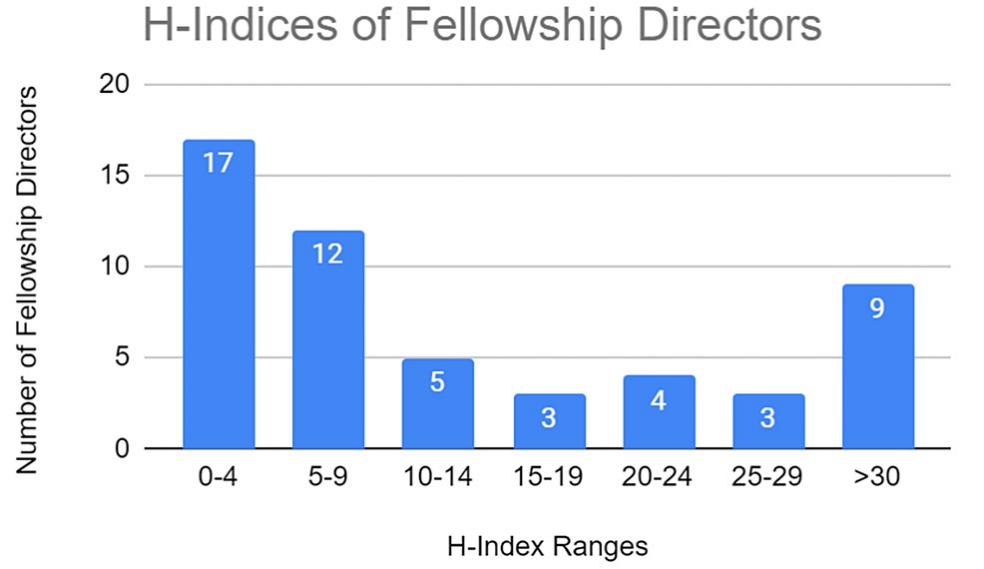
H-Indices of fellowship directors. A representation of the Scopus H-indices of all cardiothoracic radiology fellowship directors. Note: The Scopus H-indices were obtained by August 1, 2021.

Scholarly performance was assessed using the Scopus h-Index, of which data for all 53 FDs was available. A statistically significant moderate strength correlation was found between age and h-index (0.63) (Table 4). A statistically significant moderate strength correlation was found between the duration of FD appointments and the h-index (0.68) (Table 4). A statistically significant strong correlation was found between time since residency graduation and h-index (0.71) (Table 4). H-indices ranges were grouped and can be found in Figure 4. Gender-wise comparison of mean h-indices revealed 16.2 for males and 15 for females, with no significant difference between the two groups (p = 0.81). The most impactful FD had an h-index value of 84. The minimum h-index value reported for all FDs was 0.

**Table 4:** H-index correlation. Pearson correlation between variables of years working as fellowship director and Scopus h-index, fellowship director age and Scopus h-index, and years elapsed since residency graduation and Scopus h-index. Note: Regression was only calculated for datasets that included both variables. (*) indicates statistically significant values (p < 0.05).

Correlated h-indices	r (p)
Years as fellowship director vs Scopus h-index	0.68 (<0.0001)*
Age vs Scopus h-index	0.63 (<0.0001)*
Years since residency graduation vs Scopus h-index	0.71 (<0.0001)*

## Discussion

Our study summarizes the current leadership and demographic trends seen in the cardiothoracic radiology FD position. Cardiothoracic radiology FDs are often males in their late 40s. They are likely to have a strong history of research productivity with an average h-index of 15.7. We identified a large portion of them, 20/53 (37.7%), trained at the same fellowship institution where they currently hold the role of FD. There is no identifiable reason for this; however, we hypothesize that this may be due to the familiarity of the FD with the institution and vice versa.

Most cardiothoracic imaging FDs are male (58.49%). This represents a roughly 12% difference (70.6% vs 58.49%) from what was reported in an article by Purushothaman et al. that examined program directors for radiology residency [3]. Additionally, we observed from our data that female cardiothoracic FDs made up a relatively higher proportion than males in radiology as a whole [16]. Radiology as a specialty is largely made up of male physicians. The 2019 ACR Commission on Human Resources Workforce Survey showed that there has been no significant change in the percentage of females in radiology, with 2018 being the most recent update at 23% [16]. Moreover, they disclosed that there is a higher percentage of women in the younger age range, which may point to a growing inclusion in radiology in future years. West et al. reported that 22% of radiology fellows were women in 2013, which displays a relatively similar percentage of female radiologists going on to advanced radiologic training [17]. However, our research revealed females represented almost twice that with 41.5% in the leadership role of FD. This does support that a larger percentage of female radiologists take on leadership roles in academia compared to the current pool of radiologists. The proportion of female radiologists has been increasing since at least 2007 when females made up only 20.8% of radiologists [18]. Whether a similar trend is currently underway for female cardiothoracic imaging FDs will require a future study.

A study looking at gender and ethnic diversity in U.S. radiology fellowship programs, although not examining cardiothoracic imaging, showed there was a limited number of underrepresented minorities in training [17]. The reported data showed that Blacks only accounted for 3% and Hispanics accounted for 6%. Our study did not analyze trends in diversity as we did not inquire about the ethnicity of current FDs. Although the study lacked data for cardiothoracic imaging fellows, it presents the point that there should be ongoing research on gender disparities and representation. We believe that if future studies wish to be successful and accurate when examining demographics in residency or fellowship directors they should include ethnicity and race in their survey to precisely display the current statistics.

Research productivity in cardiothoracic FDs was well above the average academic radiologist in all academic ranks (i.e., instructor (2.7), assistant (2.3), associate (6.2), and full professor (12.5)) [12]. The mean h-index for FDs in this study was 15.7, with a large SD of 17. Purushothaman et al. found that the average h-index of radiology residency directors was 17, as well as a large SD of 25.6 [3]. This shows a similar h-index to our current study, which suggests a comparable level of scholarly activity is achieved by these two academic radiology subspecialties. Twenty-one of the 53 FDs had H-indices greater than 10, with a high of 84. The large distribution in the h-index could be secondary to differences in caliber, type, size of the department, and the number of fellows within the department; these factors could result in this wide range of research output. This may warrant further investigation in future studies. These findings suggest that cardiothoracic radiology fellowship programs do not value as strong of a research background as other radiology subspecialties that have been published, such as interventional neuroradiology fellowships which had a recorded h-index of 22.3 [19]. Interventional neuroradiology training can be completed through various pathways, like neurology, neurosurgery, or radiology, and requires additional years of training, which may contribute to this higher research productivity. Nevertheless, a mean h-index of 15.7 shows an impressive amount of research dedication from the cardiothoracic radiologists to achieve that.

In 2015 a study was published on change overload in radiology, which described the fatigue, frustration, and burnout felt during this time [20]. The authors concluded that this phenomenon led to the resignation of some radiology residency and fellowship program directors and therefore could influence the amount of time it takes radiology trainees to ascend through the ranks to the FD role. There is informal anecdotal evidence that job turnover for radiological program directors is disproportionately high, with a mean tenure determined to be seven years (range = 0.5-30 years) among program directors, which is identical to the data presented for cardiothoracic radiology FDs. However, job satisfaction was reported to be quite high among this population [21]. Future studies can compare the average age, time as a program director, and graduation year of other radiology fellowship directors such as in breast imaging and musculoskeletal imaging, as well as comparison to other fields of medicine.

The most attended fellowship programs among current cardiothoracic radiology FDs include the University of Maryland (5), Brigham and Women’s (4), and Massachusetts General Hospital (3). Further, the most attended residency programs include the University of Rochester (3), Albert Einstein (2), Eastern Virginia (2), Lahey Clinic (2), University of Minnesota (2), and University of Washington (2). It is not clear why the programs listed produce the most FDs. Of these, the FDs from the University of Maryland and Massachusetts General Hospital are among those with the highest h-indices among FDs. Our data would support that going to a fellowship with a high research output may pave the way to obtaining a leadership role in this field. This trend may be further due to leadership training education within these programs, or the influence of professional networks after graduation. Reputation, and possibly the generation of FDs prior to the current one, cannot be overlooked as institutional influences can open doors to FD positions. Further research is needed on this subject matter to confirm these hypotheses.

To continue with training, only one out of 53 (1.9%) of cardiothoracic FDs are DOs, whereas DOs make up 3.9% of all radiologists. The paucity of DO students has been discussed in other papers that have looked at radiology program directors [19]. This may have to do with the history of DO students not being accepted into radiology programs. However, as we transition into the single accreditation system for graduate medical education (GME), osteopathic medical students should have more access to the radiology specialty and should be better represented in the coming years.

This study has several important limitations. While we attempted to include all cardiothoracic radiology fellowship training programs, via the STR website, there is a possibility that programs without an online presence were missed in our search. Further, our group relied on publicly available data, followed by obtaining responses to surveys or CVs to establish and develop our datasets. Some CVs and other relevant information could not be obtained in some cases, while others may not be fully updated. A further consideration for this data collection method is that CVs are typically self-reported; therefore, there is a possibility of reporting bias. As a cross-sectional study, this data and research established demographics at one point in time. Results can vary year to year due to the expansion of programs, turnover of FDs, and other outside influences on FDs. This paper cannot address all the subjective factors that are embodied in particular individuals’ career paths, from professional mentors, desire to be in the FD role, and other personal factors that influence career trajectory and the hiring process. Some FD positions may not receive additional compensation other than provisions such as dedicated administrative time from their normal clinical duties and responsibilities. Thus, some may not want to add to this daily burden and responsibility in a system that does not provide added support for the role.

## Conclusions

This cross-sectional study shows the present demographics within the cardiothoracic radiology FD position. This field of radiology is observed to have FDs with research productivity that is comparable with other medical specialties. Some radiology residency and fellowship programs were shown to produce more FDs than others; however, we were not able to identify causality. Program directors appear to be selected from a familiar pool of applicants, and, ultimately, FDs are being replaced by individuals with similar distinctions. Overall, this research into cardiothoracic radiology FDs demographics and research productivity can add to the current body of literature on FDs in various medical specialties. It is essential to continue to reflect on medical leadership as the field continues to advance.
